# Gaining Insight into Exclusive and Common Transcriptomic Features Linked to Drought and Salinity Responses across Fruit Tree Crops

**DOI:** 10.3390/plants9091059

**Published:** 2020-08-19

**Authors:** Jubina Benny, Annalisa Marchese, Antonio Giovino, Francesco Paolo Marra, Anna Perrone, Tiziano Caruso, Federico Martinelli

**Affiliations:** 1 Department of Agricultural, Food and Forest Sciences, University of Palermo, Viale delle Scienze—Ed. 4, 90128 Palermo, Italy; jubina.benny@unipa.it (J.B.); tiziano.caruso@unipa.it (T.C.); 2 Council for Agricultural Research and Economics (CREA), Research Centre for Plant Protection and Certification (CREA-DC), 90011 Bagheria, Italy; antonio.giovino@crea.gov.it; 3 Department of Architecture (DARCH), University of Palermo, Viale delle Scienze—Ed. 8, 90128 Palermo, Italy; francescopaolo.marra@unipa.it; 4 Department of Biological, Chemical and Pharmaceutical Sciences and Technologies (STEBICEF), University of Palermo, Viale delle Scienze, 90128 Palermo, Italy; anna.perrone@unipa.it; 5 Department of Biology, University of Florence, Sesto Fiorentino, 50019 Florence, Italy; federico.martinelli@unifi.it

**Keywords:** abiotic stresses, transcriptomics, fruit crops, meta-analysis, RNA-seq, roots, differentially expressed genes

## Abstract

The present study aimed at identifying and mapping key genes expressed in root tissues involved in drought and salinity tolerance/resistance conserved among different fruit tree species. Twenty-six RNA-Seq samples were analyzed from six published studies in five plant species (*Olea europaea, Vitis riparia Michx, Prunus mahaleb, Prunus persica, Phoenix dactylifera*). This meta-analysis used a bioinformatic pipeline identifying 750 genes that were commonly modulated in three salinity studies and 683 genes that were commonly regulated among three drought studies, implying their conserved role in resistance/tolerance/response to these environmental stresses. A comparison was done on the genes that were in common among both salinity and drought resulted in 82 genes, of which 39 were commonly regulated with the same trend of expression (23 were upregulated and 16 were downregulated). Gene set enrichment and pathway analysis pointed out that pathways encoding regulation of defense response, drug transmembrane transport, and metal ion binding are general key molecular responses to these two abiotic stress responses. Furthermore, hormonal molecular crosstalk plays an essential role in the fine-tuning of plant responses to drought and salinity. Drought and salinity induced a different molecular “hormonal fingerprint”. Dehydration stress specifically enhanced multiple genes responsive to abscisic acid, gibberellin, brassinosteroids, and the ethylene-activated signaling pathway. Salt stress mostly repressed genes encoding for key enzymes in signaling proteins in auxin-, gibberellin-(gibberellin 2 oxidase 8), and abscisic acid-related pathways (aldehyde oxidase 4, abscisic acid-responsive element-binding protein 3). Abiotic stress-related genes were mapped into the chromosome to identify molecular markers usable for the improvement of these complex quantitative traits. This meta-analysis identified genes that serve as potential targets to develop cultivars with enhanced drought and salinity resistance and/or tolerance across different fruit tree crops in a biotechnological sustainable way.

## 1. Introduction

Drought and salinity are considered two major environmental factors affecting plant productivity and plant distribution. Therefore, it is necessary to understand plant tolerance toward drought and salinity, forming a major research topic. Drought stress represents a critical issue at reproductive stages for crop production because it impairs key physiological processes involved in yield and its components such as bud development, flowering, and fruit ripening. There are significant differences within the same species in response to drought stress, especially at the root level [1]. Drought-resistant cultivars are those that more efficiently modulate carbohydrate partitioning toward seed filling, contrasting drought stress during the pod-filling stage. It was shown that a more efficient modulation of sucrose transport favors an efficient carbon mobilization toward seeds [2]. Drought stress also reduces water uptake and affects the rapid and long-term adaptation mechanisms of plant species to climate change. Identifying the molecular mechanisms and key genes involved in drought and salinity resistance is essential for efficient next-generation molecular breeding. Plants can perceive abiotic stresses and elicit appropriate responses with altered metabolism, growth, and development. These regulatory circuits include stress sensors, signaling pathways comprising a network of protein–protein reactions, transcription factors, and hormones, and finally the output proteins or metabolites [3]. Plants are sessile organisms. Indeed, water and salt stress occur frequently, and, since plants cannot move, they developed strategies to adjust themselves with these challenges either via adaption mechanisms or via specific growth habits to avoid stress conditions. These plant cryptic way of resisting to harsh environmental stresses are modulated by a complex regulatory network that is only barely elucidated. Differential stress tolerance could be attributed to differences in plant reactivity in terms of stress perception, signal transduction and appropriate gene expression programs, or other novel metabolic pathways that are restricted to tolerant plants [4]. Exposure to drought or salt stress triggers many common reactions in plants. Both stresses cause cellular dehydration, which causes osmotic stress and water movement from the cytoplasm into the extracellular space. The stresses induce reactive oxygen species and radical ions, which in turn show a negative effect on the cellular structures and metabolism. Even though the early responses toward salinity and drought are the same, the responses toward ionic components are different. Decrease of photosynthesis or hormonal crosstalk regulation such as increased levels of abscisic acid (ABA) is a common physiological feature of both stresses. High intracellular concentrations of sodium and chloride ions are specific issues of salinity stress [5]. Over the last decade, thousands of genes involved in drought stress responses were identified, which can be included in two groups [6]. One group of genes directly protects the plants against drought stress by regulating water transport (aquaporin) [7] or by protecting the integrity of cellular membrane and macromolecules [8]. The second group of genes (receptor proteins, protein kinases, protein phosphatases, and transcription factors) regulates signal perception, signal transduction, and amplification [9]. Many key genes for salinity tolerance relating to oxidation–reduction processes, ion transport and chloride channels, hormone-related genes, like ethylene perception-related [10] and ABA [11], as well as many transcription factors, were discovered [12]. Fruit trees must exist under adverse environmental conditions over years and, therefore, require not only drought/salinity adaptiveness but also flexibility toward the metabolism of hormones, transcription factors (TF), etc. to adjust with changing conditions. For example, some key transcription factor (TF) families such as *MYB*, *WRKY*, basic leucine zipper (bZIPs) were found to be involved in a different manner depending on the type of stress [13]. Drought and salinity tolerance in fruit trees is usually achieved via biochemical modification of the cellular metabolism. [14]. Transcriptomic studies are important in identifying specific genes involved in water and salinity stresses in different species. These types of analysis help in recognizing which genes are the basis of diverse abiotic tolerance and resistance mechanisms. However, transcriptomic approaches have several drawbacks. Most transcriptomic studies are generally related to only one season, which may lead to low reliability of conclusions of these studies. RNA-Seq data are affected by high environmental variability, often presenting false-positive results. Therefore, it is necessary to adapt bioinformatic pipelines to enhance the comparison of data obtained across different species in order to strengthen the meaning of every single study and validate the published works reducing the environmental variability [15]. Meta-analysis is a statistical technique for combining the findings from independent studies. It is used to determine the effectiveness of a treatment or to study a factor affecting a process combining data from randomized similar studies. Meta-analysis provides a precise estimation of treatment’s effect giving weight to the size of the different studies included in the analysis. Meta-analysis has the power of (1) filtering the most meaningful information linked with the object of study, (2) eliminating data affected by environmental variability, (3) reducing false-positive results, and (4) increasing the number of virtual replicates. This kind of work is lacking in crops, especially at the transcriptomic level [3]. The current study is focused on fruit tree responses toward drought and salinity, as well as major genes which can be utilized by genetic engineering for the development of tolerant species. Thus, a meta-analysis of all the transcriptomic studies can play a vital role in selecting the most frequent and most significant differentially expressed genes (DEGs) among the complete list of differentially regulated genes.

In the present work, we conducted a meta-analysis by selecting six RNA-Seq studies with similar experimental design (timing and intensity of stresses) conducted in five fruit tree crops in order to deliver conserved and reliable genomic information for enhancing drought and salinity crop resistance/tolerance. We analyzed, in the most comprehensive manner possible, RNA-Seq data in fruit tree crops under drought and salinity using the same bioinformatics pipeline used in our previously published meta-analysis. The most important players among the huge amount of data generated by every single RNA-Seq study were identified and mapped on the chromosomes to develop next-generation markers (i.e., based on epigenetic mechanisms). Key molecular physiological conclusions were generated based on the identification of conserved gene sets, pathways, and gene networks involved in abiotic stress resistance/tolerance. This study provides a valid approach to ask additional questions with respect to how plants respond to stress.

## 2. Results

### 2.1. Transcriptomic Responses to Drought and Salinity

We found 12 RNA-Seq studies in public databases matching our chosen selection criteria. Among the 12 studies, six of them had no raw data available. The analysis was performed using six studies: three dealing with drought and the other three dealing with salinity.

The articles, plant species, and the number of up- and downregulated genes for each article are listed in Table 1. The analysis resulted in the identification of a total of 36,909 genes, of which 18,404 were upregulated and 18,505 were downregulated. Taking the stress-related genes toward salinity response, 51.55% of the genes were upregulated and 53.01% were downregulated. When considering the drought-related genes, 50.46% of the total number of stress-related genes were upregulated and 49.53% were downregulated.

The first comparison was performed using the three studies in salinity to find common genes regulated among them. In total, 750 genes were common among the three studies, implying their conserved role in response toward salinity. A second comparison was done comparing the three works related to drought. In total, 683 genes were common in all the three drought studies. A third comparison was done on the 683 drought-related genes and 750 salinity-related genes to find genes common among both salinity and drought. This latter comparison highlighted 82 differentially regulated genes (Table S1, Supplementary Materials) involved in drought and salinity. There were 39 genes that showed the same trend of expression: 23 were all upregulated and 16 were all downregulated. We also paid special attention to these 39 genes (Table 2) in the downstream functional analysis.

### 2.2. Gene Set and Pathway Enrichment Analysis

The Database for Annotation, Visualization and Integrated Discovery (DAVID) software was used to annotate the functionalities of genes corresponding to drought and salinity at the transcriptomic level taking the list of drought-regulated genes (common among three studies) and salinity-regulated genes (common among three studies). The functional pathways related to the drought and salinity along with gene ontology (GO) identifier, term, count, *p*-values, and Benjamini values are given in Table S2 (Supplementary Materials). Among the drought studies, two GO terms were downregulated while 13 were upregulated. It is worth mentioning some of the biological pathways that are well known to be enhanced by drought stress such as response to abscisic acid, response to jasmonic acid, defense response, protein phosphorylation, and heterochromatin maintenance. On the contrary, some GO terms that were downregulated in response to water stress were the following: response to carotenoid biosynthetic process and embryo development ending in seed dormancy. While considering the salinity responses, 14 GO terms were downregulated and 14 were upregulated. GO terms such as regulation of jasmonic acid-mediated signaling pathway, response to cadmium ion, ubiquitin-dependent protein catabolic process, cellular heat acclimation, and regulation of stomatal movement showed an enhancement toward the salinity stress, whereas leaf senescence, response to cytokinin, auxin metabolic process, late nucleophagy, transmembrane receptor protein tyrosine kinase signaling pathway, and micro autophagy of nucleus were repressed. When comparing the GO terms corresponding to each stress, no GO terms related to the biological processes were commonly downregulated among drought and salinity. On the other hand, pathways encoding regulation of defense response, transmembrane transport, and metal ion binding were enhanced toward both drought and salinity responses.

### 2.3. Transcriptomic Responses Related to Hormone Metabolism

**Figure 1:**
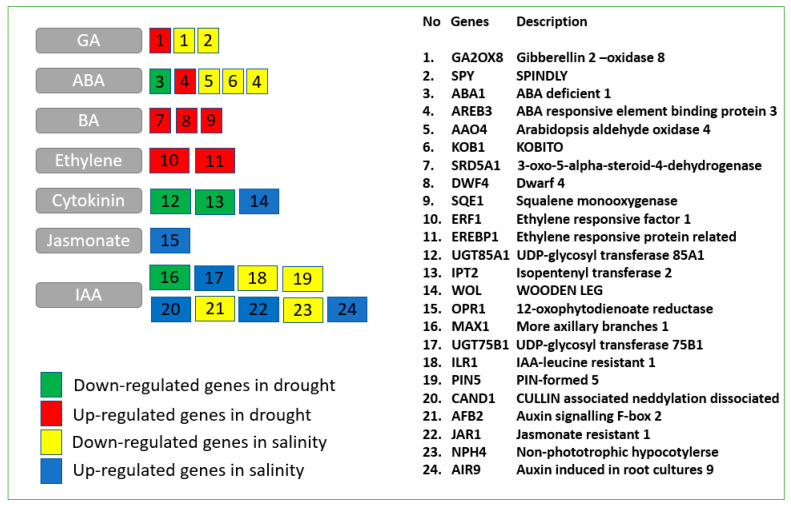
Drought- and salinity-regulated genes involved in hormone-related categories commonly regulated in the studies are shown. Genes were identified as *Arabidopsis* orthologs of each gene of the analyzed plant species. Red and green indicate the up- and down-regulated genes in drought, whereas blue and yellow indicate the up- and down-regulated genes in salinity.

We focused our attention to the hormonal-related genes considering the key role played by hormonal crosstalk in the modulation of abiotic stress responses in plants. When focusing on drought stress studies, water deprivation downregulated two genes responsive to cytokinin (uridine diphosphate (UDP)-glycosyltransferase (UGT85A1) and isopentenyl transferase 2 (IPT2)), one responsive to abscisic acid (ABA) (ABA deficient 1 (ABA1)), and one indole acetic acid (IAA) gene (more axillary branches 1 (MAX1)). On the other hand, it upregulated several genes responsive to abscisic acid, gibberellin, brassinosteroids (BRs), and ethylene-activated signaling pathway. Among the brassinosteroid-related genes, it is worth mentioning the enhancement of 3-oxo-5-alpha-steroid 4-dehydrogenase (SRD5A1), DWARF 4 (DWF4), and squalene monooxygenase (SQE1) (Figure 1).

Related to salinity stress, there was an upregulation in 12-oxophytodienoate reductase (OPR1), wooden leg (WOL), UDP-glucosyltransferase 75B1 (UGT75B1), cullin-associated and neddylation-dissociated (CAND1), jasmonate resistant 1 (JAR1), and auxin induced in root cultures 9 (AIR9). At the same time, genes encoding for auxin, gibberellins (gibberellin 2 oxidase 8 (GA2OX8)), and abscisic acid (aldehyde oxidase 4 (AO4), KOBITO 1 (KOB1), ABA-responsive element binding protein 3 (AREB3)) were downregulated.

### 2.4. Transcription Factors (TFs)

TFs are special proteins that control the transcription of genes, and many of them are, therefore, expressed in a genotype-, tissue-, and stress-specific manner. A total of 45 major TFs were differentially expressed, with 16 downregulated in both drought and salinity conditions and 23 commonly upregulated (Figure S1, Supplementary Materials). The most expressed TF families were *WRKY*, basic helix–loop–helix (bHLH), *MYB*, trihelix-factor, *APETALA2*/ethylene-responsive element binding protein (AP2-EREBP), homeobox (HB), and GATA. Among downregulated genes, it is worth mentioning ephrin type-B receptor, *WRKY2*, *O*-fructosyltransferase 1, and a zinc finger (C_3_HC_4_-type really interesting new gene (RING) finger) family protein. Among the upregulated genes, it is worth noting the expression of serine carboxypeptidase-like 51, adenosine diphosphate (ADP)-ribosylation factors-GTPase-activating proteins (ARF-GAP) domain 5, serine/threonine-protein kinase, ataxia telangiectasia mutated (ATM), and transducin/WD40 repeat-like superfamily protein, due to their involvement in plant response to abiotic stresses and ABA-dependent plant development [16].

### 2.5. Stress-Related Genes Involved in Both Drought and Salinity

**Figure 2:**
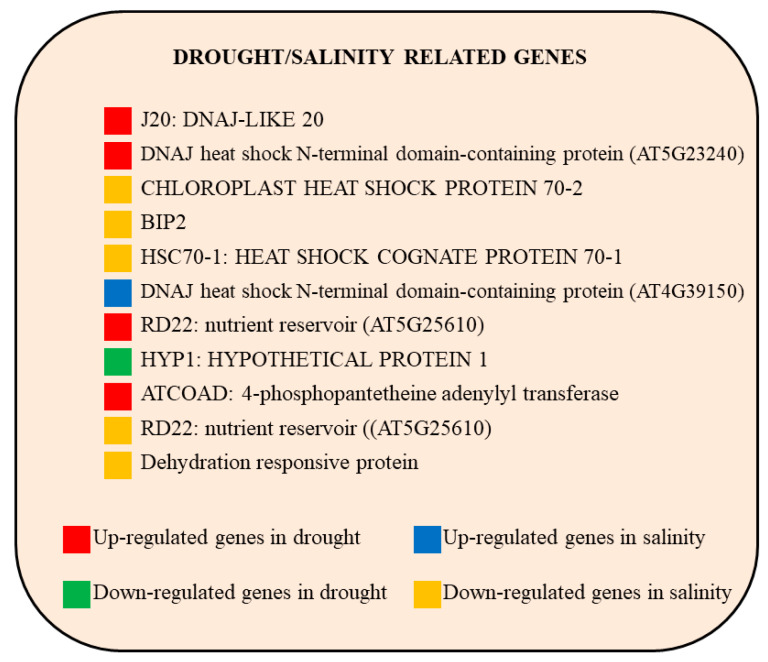
Drought/salinity-regulated genes involved in abiotic stress-related categories commonly regulated in all eight studies are indicated. Genes were identified as *Arabidopsis thaliana* orthologs of each gene of the analyzed plant species. Red indicates up-regulation and green indicates down-regulation in response to drought stress, whereas blue and yellow indicate up- and down-regulated genes in salinity.

Genes mapped to the abiotic stress (drought/salinity)-related categories were identified using MapMan, and they are shown in Figure 2**.** Among the common drought upregulated genes, it is worth mentioning the DNAJ-like 20 (J20), DNAJ heat shock N-terminal domain-containing protein, dehydration 22 (RD22) (nutrient reservoir), and 4-phosphopantetheine adenylyl transferase (ATCOAD). In the category of salt stress-related genes, we observed an upregulation of DNAJ heat shock N-terminal domain-containing protein and a downregulation of Luminal binding protein 2 (BIP2), chloroplast heat-shock protein 70-2, heat-shock cognate protein 70-1 (HSC70-1), dehydration responsive protein, and RD22.

### 2.6. Protein–Protein Interaction Network Analysis in Response to Abiotic Stresses

**Figure 3:**
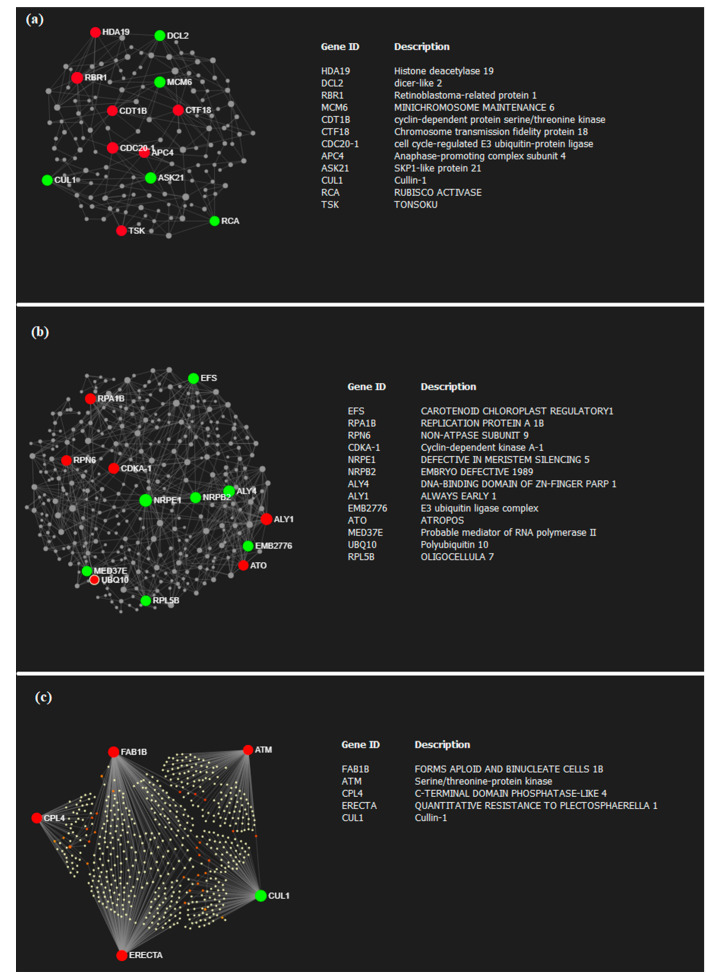
Protein–protein interaction network analysis predicted for genes commonly regulated in (**a**) three of three drought studies, and (**b**) three of three salinity studies; (**c**) genes commonly regulated in six of six studies of both drought and salinity based on *Arabidopsis* knowledgebase. Proteins encoded by genes having a high degree of betweenness are shown in red (up-regulated) and green (down-regulated).

The protein–protein interaction (PPI) network analysis comprised three networks based on the minimum default settings used to reduce the number of interacting proteins and the complexity of the networks (Figure 3). Some key genes with a high number of interactions (>20) were highlighted.

Interestingly, drought downregulated highly interactive proteins such as rubisco activase (RCA), S-phase kinase-associated protein 1 (SKP1)-like protein 21 (ASK21), and dicer-like 2 (DCL2), while it upregulated proteins such as histone deacetylase 19 (HDA19), cyclin-dependent protein serine/threonine kinase (CDT1B), retinoblastoma-related protein 1 (RBR1), and cell cycle-regulated E3 ubiquitin-protein ligase (CDC20-1) (Figure 3a). PPI network analysis was performed for salinity-regulated genes showing an enhancement of three major hub proteins, polyubiquitin 10 (UBQ10), atropos (ATO), and cyclin-dependent kinase A-1 (CDKA-1), along with a repression of embryo defective 1989 (NRPB2), DNA-binding domain of Zn-finger poly ADP ribose polymerase (PARP) 1 (ALY4), and E3 ubiquitin ligase complex (EMB2776) (Figure 3b). Among the upregulated hub (highly interacting) proteins that were present in both drought and salinity categories, it is worth noting that some proteins may play a key role in abiotic response such as forms aploid and binucleate cells 1B (FAB1B), ATM, quantitative resistance to plectosphaerella 1 (ERECTA), and one downregulated protein, cullin-1 (CUL1) (Figure 3c).

### 2.7. Genes Involved in General Dehydration Stresses

We paid special attention to the 39 (23 were upregulated and 16 were downregulated) genes showing a similar expression pattern in both drought- and salinity-related studies (Table 2). It is worth mentioning that both salinity and drought stress downregulated WRKY transcription factor 2, CUL1, nudix hydrolase 2, *O*-fructosyltransferase 1, and E3 ubiquitin-protein ligase, whereas commonly upregulated genes were phosphofructokinase 3 (PFK3), cytochrome P450 75B1, *N*-acetyl serotonin *O*-methyltransferase, ATM, and serine carboxypeptidase-like 51 (SCPL51)

These 39 key genes were mapped onto the respective chromosomes of the crops (Figure S2, Supplementary Materials). There was no homogeneous distribution of these genes across the genome observed in some of the species. The presence of a higher number of commonly regulated genes was identified in some chromosomes. In grape and olive, a homogeneous distribution of the genes in the chromosomes could be found. However, in peach, most of the genes were present in one chromosome. A total of 13 abiotic stress-related genes being mapped to chromosome 1 of peach might imply the importance of the involvement of chromosome 1 in drought and salinity resistance compared to other chromosome regions. This evidence should be taken in careful consideration by molecular breeders. This work helped in the identification of significant regions in the chromosome that contain numerous genes involved in drought and salinity. This can help guide the linking of new molecular markers capable of drought/salinity resistance.

### 2.8. Leave-One-Out Cross-Validation (LOOCV) of Meta-Analysis

We employed the LOOCV approach in order to validate the 82 hub genes identified from the study (Table S1, Supplementary Materials). This method can predict the difference between control and treated samples. We could identify a predictive accuracy of 95.03% with an area under curve (AUC) value of 0.934 (Figure S3, Supplementary Materials) for the expression levels of these genes. These results validate our meta-analysis approach to finding the hub genes responsible for the stress response.

## 3. Discussion

Roots are the first organs to be exposed to water deficiency and salt stress, and they are the first tissue to sense drought and salinity conditions. Signaling cascades transfer chemical signals toward shoots to initiate molecular responses that lead to the biochemical and morphological changes, allowing plants to be protected against water loss and salinity and to tolerate stress conditions [17]. Here, we present an overview of signaling network and gene expression regulation pathways that are actively induced in roots of fruit crops under drought and salinity stress, as these stresses are the most limiting factors of crop yield, especially in smallholder systems [18]. Although it is possible to identify a good percentage of the genetic variability due to additive genetics (major genes/alleles), probably around 20–50%, these major genes are yet to be identified. This is mainly because functional genomic studies (especially RNA-seq) present data with high variability and often contrasting evidence due to the diverse experimental conditions of studies that often escape the control of researchers. For example, in our study, we considered two studies from the same species (*Phoenix dactylifera*) but of different cultivar under salinity stress. The stress duration was different for both the studies (Table 1). While comparing the DEGs from both studies, a total of 5504 genes were identified from Yaish et al., 2017 [19], while 6676 DEGs were identified from Radwan et al., 2015 [20]. This shows that the differences in the number and type of genes modulated by stress by different cultivars are due to genotypic variance, environmental differences, different time points and stress intensity, sampling time, different growth parameters, and ways of cultivation. This is why it is important to perform meta-analyses of previously published data instead of investing more economic resources in new studies. The aim is to identify strongly associated gene loci with both drought and salt stress in order to deliver reliable molecular markers to be used in a molecular marker-assisted selection, aimed at creating new cultivars with resistance/tolerance to these strictly connected abiotic stresses. It is worth mentioning that the ongoing climate change occurring worldwide is probably affecting these two abiotic stresses more than others. The aim is to create cultivars that are beneficially responsive to multiple stresses to face the multiple harsh conditions.

### 3.1. The Role of Hormones in Drought and Salinity Responses

Our meta-analysis highlighted unexpectedly the role of hormones in complex gene regulatory networks of plant responses to abiotic stresses. Indeed, we found three genes involved in BR-related pathways that were all upregulated in response to drought (SRD5A1, DWF4, and SQE1).

BRs are polyhydroxylated steroidal hormones involved in many plant physiological processes such as hypocotyl elongation, root modulation, stomata regulation, gametophyte growth and development, and germination [21]. However, recently, their role in plant adaptation to drought was shown [22]. Plants with reduced biosynthesis of BRs are typically dwarfed and show dark green, curled leaves, small petioles, reduced hypocotyls and internodes, delayed flowering, and less fertility. On the other hand, plants with enhanced BRs show higher height and longer hypocotyls [23]. Indeed, we may speculate that the upregulation of brassinosteroids in response to drought would allow reducing the detrimental effects on key physiological processes in plants that are involved in seed production and, consequently, in crop yield. The induction of BR genes in tolerant/resistant genotypes should be taken under consideration in future validation approaches using transgenics and clustered regularly interspaced short palindromic repeats (CRISPR)-CRISPR-associated protein 9 (Cas9) technologies.

The basipetal transport of auxin was inhibited in plants under water stress, provoking losses of cotyledonary petioles and early leaf loss [24]. In addition, auxin transport inhibitors and drought had a synergistic action on leaf loss. Osmotic stress provoked a significant enhancement in the basipetal transport of auxins [25], implying a link between drought responses and polar auxin transport in plants. Contrasting evidence was observed for IAA-related genes in response to salinity; while IAA-resistant leucine 1, PIN-FORMED 5 (PIN5), *AFB2*, and Non-phototrophic hypocotyl *(NPH4)* were commonly repressed among the analyzed studies, UGT75B1, CAND1, *JAR1*, *AIR9* were upregulated. These data partially agree with previous findings showing an involvement of AIR9 and JAR1 in drought responses in barley through the action of miR2406 [26]. UGT75B1 is an auxin-related gene that controls cellular ABA content and activity through glycosylation. UGT75B1 is induced by osmotic stress, salinity, and ABA [27]. Overexpression of UGT75B1 in *Arabidopsis thaliana* provokes higher seed germination rates, larger stomatal aperture, and seedling greening in response to salt, drought, and osmotic stresses [28]. It is known that auxin plays an important role in plant growth and development. Its spatial distribution among plant tissues is modulated by polar localization of PIN-formed (PIN) auxin efflux carrier transporters, which constitute a large family [29]. The overexpression of PIN3 was shown to promote drought resistance [30]. In contrast, we found that PIN5 was downregulated by drought, implying that different members of the same family may have contrasting effects on the same abiotic stress. Several publications showed that auxin signaling plays a vital role in stress responses in plants [31], while fewer studies focused on the auxin transport response under difficult environments [25]. It is important to note that MYB, WRKY, and AP2-EREBP were highly repressed, suggesting their role in the abiotic stress response and plant growth processes [32].

The role of ethylene in drought resistance is well known [33,34]. The transgenic induction of ethylene response factor 1 (ERF1) in wheat enhanced resistance to salt and drought stress, inducing an increase in chlorophyll content, as well as superoxide dismutase and peroxidase activity [35]. These effects were probably mediated by the modulation of expression levels of some stress responsive genes [36]. ERF1 belongs to the large family of AP2/ERF genes involved in response to drought and salt stresses. However, the role of AP2/ERF genes is contrasting, since some AP2/ERF genes have negative effects such as AP23 [37]. Comparative analyses between susceptible and tolerant genotypes also confirmed a role of ERF1 in drought conditions [38]. Our meta-analysis shows an induction of ERF1 in drought conditions, agreeing with the previous evidence.

### 3.2. Key Genes and Chromosome Regions in Abiotic Stress Tolerance/Resistance

The transgenic over-expression of some *WRKY* members was shown to promote drought tolerance in *Arabidopsis thaliana* such as *WRKY20* [39]. Here, we identified a commonly upregulated *WRKY* that could be a future target for promoting drought tolerance using a transgenic approach (*WRKY6*). In addition, we found an upregulation of a transducin/WD40 repeat-like superfamily protein in response to both abiotic stresses, and this is confirmed by previous findings that showed a role of this gene in modulating ABI5 stability and abscisic acid responses in drought conditions [40]. We found that three DNAJs were commonly induced by drought, and the role of these heat-shock proteins in drought tolerance was confirmed previously. In fact, overexpression of a J-domain protein increased drought tolerance in transgenic *Arabidopsis* [41]. In addition, over-expression of *Arabidopsis* DnaJ (Hsp40) induced NaCl-stress tolerance [42]. A diverse role played by different chromosome regions in resistance to drought was already highlighted [3]. Looking at the mapping of the 39 common genes with the same trend of expression (up in both, down in both), it is possible to see that some crops showed an inhomogeneous distribution of these genes among the different chromosomes of the analyzed crop species. We found that chromosomes contained a different density of abiotic stress-related genes in peach, while, in grape and olive, their distribution seemed to be similar. In peach, 13 abiotic stress-related genes were mapped to chromosome 1. These findings highlight the need to focus on these chromosomes to develop molecular markers associated with drought and salinity resistance in these crops. Indeed, the meta-analysis showed that the mapping of the identified genes will help in understanding which genomic regions are linked to abiotic stress resistance, helping the development of sustainable breeding strategies based on next-generation molecular markers.

### 3.3. A Hypothetical Transductional Signal in Response to Osmotic Stresses

**Figure 4:**
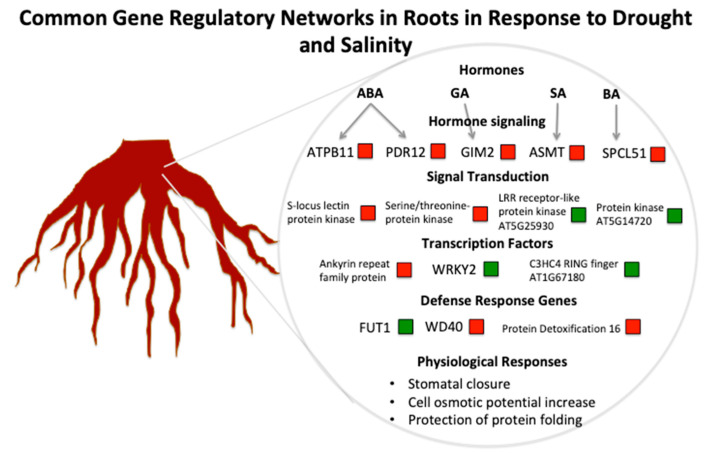
Main gene regulatory networks in common between responses to drought and salinity. Key genes involved in hormonal signaling, transduction signal, transcription regulation, and defense responses identified by the meta-analysis are indicated together with physiological effects. Upregulated genes are shown in red, while downregulated genes are shown in green.

The discovery of common features between the two types of osmotic stress in the transductional signal at the transcript level will allow the identification of reliable target genes that play a key role in drought/salinity tolerance/resistance. The role of 39 common genes in gene regulatory networks in response to general osmotic stress is shown in Figure 4. Five genes, involved in hormone signaling, were up-regulated: ATB11 and ATP-dependent permease (PDR12) (ABA), germination insensitive to ABA mutant 2 (GIM2) (gibberellins), acetyl serotonin *O*-methyltransferase (ASMT) (salicylic acid), and SPCL51 (brassinosteroids). There is previous evidence that these genes are involved in drought or salinity tolerance/resistance [43,44,45]. PDR12 is a PDR-type ABC transporter that mediates cellular uptake of abscisic acid, and mutant experiments demonstrated that this gene facilitates stomata closure and enhances drought tolerance [44]. Plants over-expressing a member of the same ABC transporter family showed increased resistance to drought and salt stress [46]. In the same way, over-expression of ASMT was shown to improve drought tolerance in *Arabidopsis thaliana* [47]. GIM2 enhanced GA biosynthesis while inhibiting ABA biosynthesis. GIM2 mutant seeds showed an ABA-insensitive phenotype during the germination and post-germination stage [48]. A serine carboxypeptidase was shown to regulate BRI1 involved in brassinosteroid signaling [49], while another member of the same family is involved in brassinosteroid-mediated responses to both biotic and abiotic stresses [49]. Related to signal transduction of drought/salinity stress, the following kinases were commonly modulated among drought- and salinity-related studies: S-locus lectin protein kinase, serine, LRR receptor-like protein kinase (AT5G25930), and protein kinase (AT5G14720). Among transcription factors, it is worth mentioning ankyrin repeat family protein (upregulated), *WRKY2* (downregulated), and C2HC4 RING finger (AT5G14720) (downregulated). Ankyrin repeat family members were also shown to be modulated by drought [50], and a RING zinc finger ankyrin protein was isolated and characterized from drought-tolerant *Artemisia desertorum* [51]. Interestingly, we found that *WRKY2* was repressed by drought and salinity, and this was unexpected since these genes were shown to be induced by NaCl and mannitol stress. This gene is a nuclear-localized transcription factor, and its role in osmotic stress needs to be clarified [52]. Nevertheless, studies showed that the expression of *WRKY2* gene in *Poncirus trifoliata* was suppressed by 27−50% upon exposure to prolonged drought stress [53]. Moreover, the expression of this gene increased initially when both cold-tolerant *Poncirus* and cold-sensitive *Citrus maxima* (pummelo) were exposed to cold stress. However, the gene expression subsided in both cold-tolerant *Poncirus* and cold-sensitive pummelo after exposure to 1 h and one day of cold stress, respectively [54]. The reason behind the repression of WRKY2 in the analysis could be the duration of the stress (14–90 DAS) selected for the study. Among the defense response genes, we identified WD40D, which, in wheat, functions as a positive regulator of salt stress and osmotic stress responses. This evidence was demonstrated by the downregulation of TaWD40D through virus-induced gene silencing, which provoked a decrease in relative water content and reduced growth compared to non-silenced lines. Indeed, it was already hypothesized that this gene might be used for the genetic improvement of stress tolerance in crop plants [55]. In addition, a fructosyl transferase (FUT) was linked with an increased tolerance to osmotic stress in *Pyropia tenera* [56], and our data confirmed this evidence. The upregulation of protein detoxification 16 may be explained by the well-known fact that the upregulation of detoxification processes generally drives enhanced resistance to abiotic stresses, linked to increased radical ions and highly reactive oxygen species [57].

In conclusion, we believe that the information provided by this work may be useful in developing molecular markers linked to these 39 genes or at least a subset of them (Figure 4); moreover, this study can facilitate targeting them with innovative biotechnological tools (transgenesis, genome editing) to create genotypes with enhanced resistance to drought/salinity stress resistance in crops. Studies confirmed that the abiotic stress-related genes identified in this study can be selected as molecular markers usable for the improvement of these complex quantitative traits [58]. This meta-analysis identified genes serving as potential targets for molecular breeding activities to develop cultivars with enhanced drought and salinity resistance and tolerance across different crops in a biotechnologically sustainable way.

## 4. Materials and Methods

### 4.1. Search Strategy for Selection of RNA-Seq Studies

For the analysis, the most relevant articles on drought and salinity stress response in fruit crops, together with one herbaceous species, were taken into consideration. These studies, identified from Scopus and PubMed, were considered suitable when abiding by the following three criteria: (i) presenting RNA-Seq sequencing methodology; (ii) mentioning at least one of the following terms in title and abstract: drought, salinity, root, stress, and abiotic stress; (iii) the presence of publicly accessible raw data. These criteria were met in six articles on a total of 26 samples (Table 3).

The selected studies were grouped based on stress: three articles were focused on drought and three articles were focused on salinity. For the functional analysis, the following groups were considered:

(A) Commonly regulated genes among three articles in drought.
(B) Commonly regulated genes among three articles in salinity.
(C) Commonly regulated genes among both (A) and (B).

**Figure 5:**
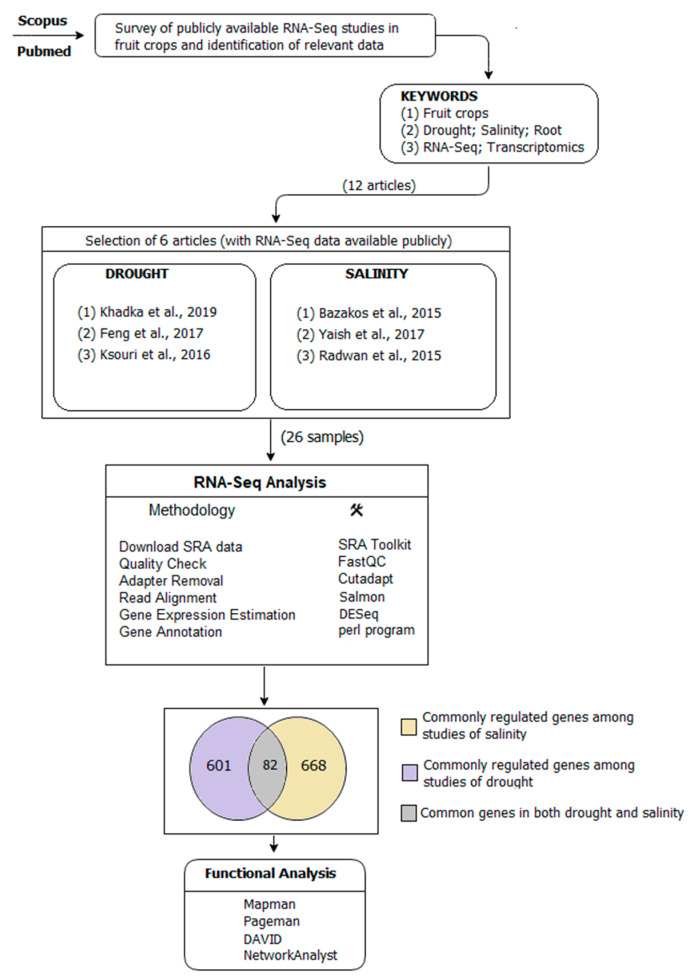
Workflow of the meta-analysis of the six transcriptomic studies related to drought and salinity stress in root tissue. Functional and statistical data analysis are indicated.

The entire workflow of the study is given in Figure 5.

### 4.2. Read Alignment, Gene Differential Expression, and Annotation

For each of the six articles, the relative crop genome and the annotation file were downloaded from Phytozome (https://phytozome.jgi.doe.gov) and the National Center for Biotechnology Information (NCBI) (https://www.ncbi.nlm.nih.gov). According to the accession number provided in the selected articles, raw data were downloaded from the NCBI sequence read archive (SRA) (https://www.ncbi.nlm.nih.gov/sra) and European Molecular Biology Laboratory (EMBL) ArrayExpress (https://www.ebi.ac.uk/arrayexpress/). The raw data were then converted to FASTQ format using SRA toolkit version 2.3.5. We trimmed low-quality bases and adapter sequences from the raw data using Cutadapt version 1.8.1 to obtain high-quality clean reads. These reads were aligned to the corresponding genome using Salmon version 0.14.0 with default parameters. To aggregate the transcript-level quantification to the gene level for gene-level differential expression analysis, we used the R package called tximport. The quantification results of salmon were then given to DESeq2 for the differential expression analysis. Up- and downregulated genes with *p*-value < 0.05, log2FC ≤ −2, and log2FC ≥ 2 were considered for downstream functional analysis. The statistical tests were corrected using the Benjamini–Hochberg false discovery rate (FDR) procedure with the help of the *p*.adjust function of R. The annotation of DEGs selected was performed using the related crop genome mapping files retrieved from the Phytozome database. For the selection of genes and their genome, along with chromosome mapping, a custom-made in-house Perl script was employed.

### 4.3. Statistical and Cluster Analysis

The DEGs corresponding to each independently studied research work, having a *p*-value < 0.05, were then analyzed by undertaking appropriate statistical tests, corrected for multiple comparisons using the p.adjust function of R and FDR [59]. By adjusting the *p*-values, the false discovery rate (FDR) was selected to a desired level of α = 0.01. Sample normalization was adopted in order to avoid systematic variation among the studies selected for the meta-analysis. The normalization served as a crucial and rigorous pre-processing step to adjust the sequencing depths and technical effects. Geometric normalization was used, whereby fragments per kilobase of transcript per million (FPKM) and fragment counts were scaled via the median of the geometric means of fragment counts across all libraries, as described in Reference [60]. R software was used for all statistical analyses. The dendrogram was constructed using Euclidean distance measure for identifying the clustering patterns of the examined drought and salinity studies (Figure S4, Supplementary Materials).

### 4.4. Gene Set and Pathway Enrichment Analysis

All the DEGs from each study were taken and aligned to the *Arabidopsis thaliana* reference genome for obtaining the best hit “The Arabidopsis Information Resource” (TAIR) ID. MapMan [61] (http://mapman.gabipd.org/) was used for mapping and the visualization of key metabolic pathways such as secondary metabolism, hormone regulation, transcription factors, and protein targeting using the *Arabidopsis thaliana* mapping file. The drought-regulated genes common in three of three studies were visualized first; then, the common salinity-regulated genes among the three studies in the salinity group and, at last, the common genes between drought and salinity stresses were visualized. Differences among metabolic pathways were visualized by the PageMan [62] analysis, a plugin of MapMan, by means of the Wilcoxon test algorithm, without any correction, using an over-representation analysis (ORA) cut-off value of 3. The TAIR IDs produced from the analysis of the each group were searched against the DAVID [63] version 6.8 Web server (https://david.ncifcrf.gov/). The information related to biological process, cellular component, and molecular function were retrieved from the GO result.

### 4.5. Mapping of Genes to Corresponding Chromosomes

The chromosome mapping was done by selecting the commonly regulated abiotic stress-related genes involved in both drought and salinity. With the help of a custom-made Perl script, we fetched chromosome number along with the start and end of the commonly regulated gene IDs, and then we located the chromosome number, with start and endpoints of each species accordingly.

### 4.6. Protein–Protein Interaction Network

NetworkAnalyst [64], a web-based tool for network-based visual analytics for gene expression profiling, meta-analysis, protein–protein interaction network analysis, and visual exploration, was used for individual data annotation and analysis. The list of homologous TAIR IDs from three groups was uploaded separately and mapped against the Search Tool for the Retrieval of Interacting Genes/Proteins (STRING) interactome database with default parameters (confident score cut off  =  900 and with experimental evidence) provided in NetworkAnalyst. To study the key connectives and to simplify the large network, we selected the “Minimum Network” setting provided by STRING. Networks were modified indicating if genes were up- or downregulated in response to each stress.

### 4.7. Validation Analysis

We implemented a leave-one-out cross-validation (LOOCV) methodology for validating the expression value of the 82 common (hub) genes. The dataset was split into two: a training set and a test set for the validation. We discarded one sample from the main dataset for testing and selected the others for training.

## Figures and Tables

**Table 1:** The number of upregulated and downregulated genes in response to drought/salinity for each study; those commonly regulated in drought, salinity, and both drought and salinity are given.

Articles	Crops	Sample Information		
		Total	Down	Up
**Drought**				
Khadka et al. 2019	*Vitis riparia* Michx	5021	2950	2071
Feng et al. 2017	*Prunus mahaleb* L.	6959	3056	3903
Ksouri et al. 2016	*Prunus persica*	5856	2829	3027
**Salinity**				
Bazakos et al. 2015	*Olea europaea* L. cv. Kalamon	6060	2982	3911
Yaish et al. 2017	*Phoenix dactylifera* L. cv. Khalas	5504	3585	1919
Radwan et al. 2015	*Phoenix dactylifera* cv. Deglet Beida	6676	3103	3573
Commonly regulated in drought		683	349	334
Commonly regulated in salinity		750	390	360
Commonly regulated among both drought and salinity		39	16	23
Common genes among drought and salinity		82		

**Table 2:** Comparison highlighting 39 genes with the same trend of expression among drought and salinity (23 were all upregulated and 16 were all downregulated). The gene identifier (ID), expression type, description, functional term, and category are given.

Gene ID	Description	Functional Term	Functional Category
**Down-Regulated Genes**			
AT5G11700	Ephrin type-B receptor ephrin type-B receptor	Vacuole	Cellular component
AT5G56270	Probable WRKY transcription factor 2	DNA-binding transcription factor activity	Molecular function
AT1G15060	Alpha/beta hydrolase family protein	Hydrolase activity	Molecular function
AT1G04910	*O*-Fructosyl transferase 1	Carbohydrate metabolic process	Biological process
AT5G24090	Acidic endo chitinase	Chitin catabolic process	Biological process
AT1G67180	Zinc finger (C_3_HC_4_-type RING finger) protein	Cell cycle	Biological process
AT3G61790	E3 ubiquitin protein ligase	Ubiquitin-dependent protein catabolic process	Biological process
AT4G02570	Cullin 1 (CUL1) AT4G02570 protein	Auxin-activated signaling pathway	Biological process
AT2G42520	DEAD-box adenosine triphosphate (ATP)-dependent RNA helicase 37	Nucleic acid binding	Molecular function
AT5G47650	Nudix hydrolase 2	Metal ion binding	Molecular function
AT5G25930	Leucine-rich repeat receptor-like protein kinase	Signaling receptor kinase	Biological process
AT4G32010	B3 domain transcription repressor; Viviparous-1/Abscisic acid insensitive 3-like2 (VAL2)	Regulation of transcription, DNA-templated	Biological process
AT3G19840	Pre-mRNA-processing protein 40C	Messenger RNA (mRNA) processing	Biological process
AT2G27900	Ras-related protein (RABA)5d	Endocytic recycling	Biological process
AT5G14720	Protein kinase superfamily protein	Phosphorylation	Biological process
AT4G32850	Polynucleotide adenylyl transferase 4	Nucleotidyltransferase activity	Molecular function
**Up-Regulated Genes**			
AT4G03500	Ankyrin repeat family protein	Membrane	Cellular component
AT1G15520	Pleiotropic drug resistance 12	Abscisic acid transport	Biological process
AT1G02520	ATP-binding cassette B11	Transmembrane transport	Biological process
AT3G06880	Transducin/ tryptophan-aspartic (WD)40 repeat-like superfamily protein	Response to stress	Biological process
AT5G64813	Light insensitive period 1	Cytoplasm	Cellular component
AT4G31210	DNA topoisomerase, type IA	Metal ion binding	Molecular function
AT5G07990	Cytochrome P450 75B1	Oxidation–reduction process	Biological process
AT5G52450	Protein detoxification 16	Response to nematode	Biological process
AT2G36690	Germination insensitive to ABA mutant 2 (GIM2)	Oxidation–reduction process	Biological process
AT5G11040	vascular network defective 4 (VAN4)	Cytokinesis by cell plate formation	Biological process
AT5G23150	Enhancer of AG-4 2 (HUA2)	Regulation of transcription by RNA polymerase II	Biological process
AT3G14270	forms aploid and binucleate cells 1B (FAB1B)	Phosphatidylinositol phosphorylation	Biological process
AT2G03810	18S pre-ribosomal assembly gar2-like protein	Regulation of asymmetric cell division	Biological process
AT4G26270	phosphofructokinase 3 (PFK3)	Fructose 6-phosphate metabolic process	Biological process
AT5G58003	C-terminal domain phosphatase-like 4	Dephosphorylation of RNA polymerase II C-terminal domain	Biological process
AT4G35160	*N*-Acetyl serotonin *O*-methyl transferase	Methylation	Biological process
AT2G45550	Cytochrome P450	Oxidation–reduction process	Biological process
AT2G19130	S-locus lectin protein kinase family protein	Phosphorylation	Biological process
AT4G02590	unfertilized embryo sac 12 (UNE12)	Regulation of defense response	Biological process
AT3G48190	Serine/threonine protein kinase	DNA damage checkpoint	Biological process
AT5G54310	ARF-GAP domain 5 (AGD5)	Activation of GTPase activity	Biological process
AT2G26330	Quantitative resistance to plectosphaerella 1	Receptor Serine/Threonine Kinase Binding	Molecular function
AT2G27920	serine carboxypeptidase-like 51 (SCPL51)	Proteolysis	Biological process

**Table 3:** Articles, crops, number of samples, stress, and sample description (control vs. treatment) included in the analysis.

Article	Stress	Species	No. of Samples	Control	Treated	Duration of Stress
Khadka et al. 2019	Drought	*Vitis riparia* Michx	6	SRR6494883 (Control1)	SRR6494880 (Treated 1)	Roots were harvested at 14 days after stress (DAS)
				SRR6494884 (Control2)	SRR6494881 (Treated 2)	
				SRR6494885 (Control3)	SRR6494882 (Treated 3)	
Feng et al. 2017	Drought	*Prunus mahaleb* L.	6	SRR5112808 (Control1)	SRR5112805 (Treated1)	Roots were harvested at 15 DAS
				SRR5112809 (Control2)	SRR5112806 (Treated2)	
				SRR5112810 (Control3)	SRR5112807 (Treated3)	
Ksouri et al. 2016	Drought	*Prunus persica*	6	SAMEA3861653 (Control 1)	SAMEA3861656 (Treated1)	Roots were harvested at 16 DAS
				SAMEA3861654 (Control2)	SAMEA3861657 (Treated2)	
				SAMEA3861655 (Control3)	SAMEA3861658 (Treated3)	
				SRR6770841 (Control 2)	SRR6770840 (Treated 2)	
Bazakos et al. 2015	Salinity	*Olea europaea* L. cv. Kalamon	2	SRR891235 (Control1)	SRR886308 (Treated1)	Roots were harvested at 90 DAS
Yaish et al. 2017	Salinity	*Phoenix dactylifera* L. cv. Khalas	2	SRR4034943 (Control1)	SRR4034944 (Treated1)	Roots were harvested at 45 DAS
Radwan et al. 2015	Salinity	*Phoenix dactylifera* L. cv. Deglet Beida	4	SRR2027988 (Control 1)	SRR2029376 (Treated 1)	Roots were harvested at 60 DAS
				SRR2029378 (Control 2)	SRR2029381 (Treated 2)	

## Supplementary Materials

The following are available online at https://www.mdpi.com/2223-7747/9/9/1059/s1. Figure S1: Drought/salinity-regulated genes involved in transcription factors which are commonly regulated in the studies are shown. Genes were identified as Arabidopsis thaliana orthologs of each gene of the analyzed plant species. Red indicates up-regulation and green indicates down-regulation in response to drought whereas blue indicates up-regulation and yellow indicates down-regulation in response to salinity, Figure S2: The 39 commonly regulated key genes that were drought/salinity-regulated in six of six studies are mapped on the respective chromosomes of the crops, Figure S3: Validation analysis. (A) ROC curve for the 82 genes. (B) The predictive accuracy (green slice) and error (red slice) rate of LOOCV for the 82 genes, Figure S4: Dendrogram showing the hierarchical relationship among the RNA-Seq studies selected for the drought/salinity stress analysis. Resulting log2FC values of the analysis were used for generating the tree. Plant species used for the analysis (six studies) are indicated, Table S1: Comparison highlighting 82 commonly modulated genes among drought and salinity; 39 showed the same trend of expression (23 were all upregulated and 16 were all downregulated). Red and green show the commonly down- and upregulated genes, respectively, Table S2: Functional pathways regulated by commonly regulated genes in drought and salinity along with GO ID, GO term, count, p-values, and Benjamini values obtained from DAVID software.

## References

[1] Lynch J.P. (2018). Rightsizing root phenotypes for drought resistance. J. Exp. Bot..

[2] Cuellar-Ortiz S.M., Arrieta-Montiel M.D.L.P., Acosta-Gallegos J., Covarrubias A.A. (2008). Relationship between carbohydrate partitioning and drought resistance in common bean. Plant Cell Environ..

[3] Benny J., Pisciotta A., Caruso T., Martinelli F. (2019). Identification of key genes and its chromosome regions linked to drought responses in leaves across different crops through meta-analysis of RNA-Seq data. BMC Plant Biol..

[4] Zhu J.K. (2001). Plant salt tolerance. Trends Plant Sci..

[5] Flowers T.J., Colmer T.D. (2015). Plant salt tolerance: Adaptations in halophytes. Ann. Bot..

[6] Meng H.L., Zhang W., Zhang G.H., Wang J.J., Meng Z.G., Long G.Q., Yang S.C. (2018). Unigene-based RNA-seq provides insights on drought stress responses in *Marsdenia tenacissima*. PLoS ONE.

[7] Alexandersson E., Danielson J.A., Rade J., Moparthi V.K., Fontes M., Kjellbom P., Johanson U. (2010). Transcriptional regulation of aquaporins in accessions of *Arabidopsis* in response to drought stress. Plant J..

[8] Golldack D., Li C., Mohan H., Probst N. (2014). Tolerance to drought and salt stress in plants: Unraveling the signaling networks. Front. Plant Sci..

[9] Shinozaki K., Yamaguchi-Shinozaki K., Seki M. (2003). Regulatory network of gene expression in the drought and cold stress responses. Curr. Opin. Plant Biol..

[10] Ruiz K.B., Maldonado J., Biondi S., Silva H. (2019). RNA-seq Analysis of Salt-Stressed Versus Non Salt-Stressed Transcriptomes of *Chenopodium quinoa* Landrace R49. Genes.

[11] Osakabe Y., Yamaguchi-Shinozaki K., Shinozaki K., Tran L.S.P. (2013). ABA control of plant macro element membrane transport systems in response to water deficit and high salinity. New Phytol..

[12] Zhang J., Jiang D., Liu B., Luo W., Lu J., Ma T., Wan D. (2014). Transcriptome dynamics of a desert poplar (*Populus pruinosa*) in response to continuous salinity stress. Plant Cell Rep..

[13] Hoang X.L.T., Nhi D.N.H., Thu N.B.A., Thao N.P., Tran L.S.P. (2017). Transcription Factors and Their Roles in Signal Transduction in Plants under Abiotic Stresses. Curr. Genom..

[14] Chen X., Lin S., Liu Q., Huang J., Zhang W., Lin J., Wang Y., Ke Y., He H. (2014). Expression and interaction of small heat shock proteins (sHsps) in rice in response to heat stress. Biochim. Biophys. Acta (BBA) Proteins Proteom..

[15] Benny J., Perrone A., Marra F.P., Pisciotta A., Caruso T., Martinelli F. (2019). Deciphering transcriptional regulation mechanisms underlining fruit development and ripening in *Vitis vinifera*. J. Berry Res..

[16] Kulik A., Wawer I., Krzywińska E., Bucholc M., Dobrowolska G. (2011). SnRK2 Protein Kinases—Key Regulators of Plant Response to Abiotic Stresses. OMICS: Int. J. Integr. Biol..

[17] Kwasniewski M., Daszkowska-Golec A., Janiak A., Chwialkowska K., Nowakowska U., Sablok G., Szarejko I. (2015). Transcriptome analysis reveals the role of the root hairs as environmental sensors to maintain plant functions under water-deficiency conditions. J. Exp. Bot..

[18] Polania J.A., Poschenrieder C., Beebe S., Rao I.M. (2016). Effective Use of Water and Increased Dry Matter Partitioned to Grain Contribute to Yield of Common Bean Improved for Drought Resistance. Front. Plant Sci..

[19] Yaish M.W., Patankar H.V., Assaha D.V.M., Zheng Y., Al-Yahyai R., Sunkar R. (2017). Genome-wide expression profiling in leaves and roots of date palm (*Phoenix dactylifera* L.) exposed to salinity. BMC Genom..

[20] Radwan O., Arro J., Keller C., Korban S.S. (2015). RNA-Seq Transcriptome Analysis in Date Palm Suggests Multi-Dimensional Responses to Salinity Stress. Trop. Plant Biol..

[21] Rozhon W., Akter S., Fernandez A., Poppenberger B. (2019). Inhibitors of Brassinosteroid Biosynthesis and Signal Transduction. Molecules.

[22] Fàbregas N., Lozano-Elena F., Blasco-Escámez D., Tohge T., Martínez-Andújar C., Albacete A., Osorio S., Bustamante M., Riechmann J.L., Nomura T. (2018). Overexpression of the vascular brassinosteroid receptor BRL3 confers drought resistance without penalizing plant growth. Nat. Commun..

[23] Nie S., Huang S., Wang S., Cheng D., Liu J., Lv S., Li Q., Wang X. (2017). Enhancing Brassinosteroid Signaling via Overexpression of Tomato (*Solanum lycopersicum*) SlBRI1 Improves Major Agronomic Traits. Front. Plant Sci..

[24] Davenport R.J., Reid R.J., Smith F.A. (1997). Sodium-calcium interactions in two wheat species differing in salinity tolerance. Physiol. Plant..

[25] Zhang Q., Li J., Zhang W., Yan S., Wang R., Zhao J., Li Y., Qi Z., Sun Z., Zhu Z. (2012). The putative auxin efflux carrier OsPIN3 tis involved in the drought stress response and drought tolerance. Plant J..

[26] Fard E.M., Bakhshi B., Keshavarznia R., Nikpay N., Shahbazi M., Salekdeh G.H. (2017). Drought responsive microRNAs in two barley cultivars differing in their level of sensitivity to drought stress. Plant Physiol. Bioch..

[27] Chen T.T., Liu F.F., Xiao D.W., Jiang X.Y., Li P., Zhao S.M., Hou B.K., Li Y.J. (2020). The *Arabidopsis* UDP-glycosyltransferase75B1, conjugates abscisic acid and affects plant response to abiotic stresses. Plant Mol. Biol..

[28] Dalal M., Sahu S., Tiwari S., Rao A.R., Gaikwad K. (2018). Transcriptome analysis reveals interplay between hormones, ROS metabolism and cell wall biosynthesis for drought-induced root growth in wheat. Plant Physiol. Bioch..

[29] Zhang K.X., Xu H.H., Yuan T.T., Zhang L., Lu Y.T. (2013). Blue-light-induced PIN3 polarization for root negative phototropic response in *Arabidopsis*. Plant J..

[30] Tognetti V.B., Aken O.V., Morreel K., Vandenbroucke K., Cotte B.V.D., Clercq I.D., Chiwocha S., Fenske R., Prinsen E., Boerjan W. (2010). Perturbation of Indole-3-Butyric Acid Homeostasis by the UDP-Glucosyltransferase UGT74E2 Modulates *Arabidopsis* Architecture and Water Stress Tolerance. Plant Cell.

[31] Benny J., Marra F.P., Giovino A., Balan B., Caruso T., Martinelli F., Marchese A. (2020). Transcriptome Analysis of *Pistacia vera* Inflorescence Buds in Bearing and Non-Bearing Shoots Reveals the Molecular Mechanism Causing Premature Flower Bud Abscission. Genes.

[32] Ksouri N., Jiménez S., Wells C.E., Contreras-Moreira B., Gogorcena Y. (2016). Transcriptional Responses in Root and Leaf of *Prunus persica* under Drought Stress Using RNA Sequencing. Front. Plant Sci..

[33] Khadka V.S., Vaughn K., Xie J., Swaminathan P., Ma Q., Cramer G.R., Fennell A.Y. (2019). Transcriptomic response is more sensitive to water deficit in shoots than roots of *Vitis riparia* (Michx.). BMC Plant Biol..

[34] Amirbakhtiar N., Ismaili A., Ghaffari M.R., Firouzabadi F.N., Shobbar Z.-S. (2019). Transcriptome response of roots to salt stress in a salinity-tolerant bread wheat cultivar. PLoS ONE.

[35] Xing L., Zhao Y., Gao J., Xiang C., Zhu J.K. (2016). The ABA receptor PYL9 together with PYL8 plays an important role in regulating lateral root growth. Sci. Rep..

[36] Wang H., Wang H., Shao H., Tang X. (2016). Recent Advances in Utilizing Transcription Factors to Improve Plant Abiotic Stress Tolerance by Transgenic Technology. Front. Plant Sci..

[37] Zhuang J., Chen J.M., Yao Q.H., Xiong F., Sun C.C., Zho X.R., Zhang J., Xiong A.S. (2010). Discovery and expression profile analysis of AP2/ERF family genes from *Triticum aestivum*. Mol. Biol. Rep.

[38] Deokar A.A., Kondawar V., Jain P.K., Karuppayil S.M., Raju N.L., Vadez V., Varshney R.K., Srinivasan R. (2011). Comparative analysis of expressed sequence tags (ESTs) between drought-tolerant and -susceptible genotypes of chickpea under terminal drought stress. BMC Plant Biol..

[39] Feng Y., Liang C., Li B., Wan T., Liu T., Cai Y. (2017). Differential expression profiles and pathways of genes in drought resistant tree species Prunus mahaleb roots and leaves in response to drought stress. Sci. Hortic..

[40] Wan X., Zou L.H., Zheng B.Q., Wang Y. (2019). Circadian Regulation of Alternative Splicing of Drought-Associated CIPK Genes in *Dendrobium catenatum* (Orchidaceae). Int. J. Mol. Sci..

[41] Xia J., Kong D., Xue S., Tian W., Li N., Bao F., Hu Y., Du J., Wang Y., Pan X. (2014). Nitric oxide negatively regulates AKT1-mediated potassium uptake through modulating vitamin B6 homeostasis in Arabidopsis. Proc. Natl. Acad. Sci. USA.

[42] Zhao Z., Zhang W., Yan J., Zhang J., Liu Z.L.X., Yi Y. (2010). Over-expression of Arabidopsis DnaJ (Hsp40) contributes to NaCl-stress tolerance. Afr. J. Biotechnol..

[43] Bazakos C., Manioudaki M.E., Sarropoulou E., Spano T., Kalaitzis P. (2015). 454 Pyrosequencing of Olive (*Olea europaea* L.) Transcriptome in Response to Salinity. PLoS ONE.

[44] Kang J., Hwang J.U., Lee M., Kim Y.Y., Assmann S.M., Martinoia E. (2010). PDR-type ABC transporter mediates cellular uptake of the phytohormone abscisic acid. Proc. Natl. Acad. Sci. USA.

[45] Khan S., Anwar S., Yu S., Sun M., Yang Z., Gao Z.Q. (2019). Development of Drought-Tolerant Transgenic Wheat: Achievements and Limitations. Int. J. Mol. Sci..

[46] Kim S., Kang J., Cho D., Park J., Kim S. (2004). ABF2, an ABRE-binding bZIP factor, is an essential component of glucose signaling and its overexpression affects multiple stress tolerance. Plant J..

[47] Zuo B., Zheng X., He P., Wang L., Lei Q., Feng C., Zhou J., Li Q., Han Z., Kong J. (2014). Overexpression of MzASMT improves melatonin production and enhances drought tolerance in transgenic *Arabidopsis thaliana* plants. J. Pineal Res..

[48] Xiong W., Ye T., Yao X., Liu X., Ma S., Chen X., Chen M.L., Feng Y.Q., Wu Y. (2018). The dioxygenase GIM2 functions in seed germination by altering gibberellin production in *Arabidopsis*. J. Integr. Plant Biol..

[49] Li J., Nam K.H., Vafeados D., Chory J. (2001). BIN2, a New Brassinosteroid-Insensitive Locus in Arabidopsis. Plant Physiol..

[50] Sakamoto H., Matsuda O., Iba K. (2008). ITN1, a novel gene encoding an ankyrin-repeat protein that affects the ABA-mediated production of reactive oxygen species and is involved in salt-stress tolerance in *Arabidopsis thaliana*. Plant J..

[51] Yang X., Sun C., Hu Y., Lin Z. (2008). Molecular cloning and characterization of a gene encoding RING zinc finger ankyrin protein from drought-tolerant *Artemisia desertorum*. J. Biosci..

[52] Jiang W., Yu D. (2009). Arabidopsis WRKY2 transcription factor mediates seed germination and post germination arrest of development by abscisic acid. BMC Plant Biol..

[53] Banerjee A., Roychoudhury A. (2015). WRKY Proteins: Signaling and Regulation of Expression during Abiotic Stress Responses. Sci. World J..

[54] Şahin-Çevik M. (2012). A WRKY transcription factor gene isolated from *Poncirus trifoliata* shows differential responses to cold and drought stresses. Plant Omics.

[55] Kong D., Li M., Dong Z., Ji H., Li X. (2014). Identification of TaWD40D, a wheat WD40 repeat-containing protein that is associated with plant tolerance to abiotic stresses. Plant Cell Rep..

[56] Wi J., Jung H.S., Im S., Yang S., Park E.J., Hwang M.S., Jeong W.J., Choi D.W. (2017). A nuclear fructosyltransferase-like protein, PtFUT, from marine red alga *Pyropia tenera* (Rhodophyta) confers osmotic stress tolerance. J. Appl. Phycol..

[57] Hossain M.A., Bhattacharjee S., Armin S.M., Qian P., Xin W., Li H.Y., Burritt D.J., Fujita M., Tran L.S.P. (2015). Hydrogen peroxide priming modulates abiotic oxidative stress tolerance: Insights from ROS detoxification and scavenging. Front. Plant Sci..

[58] Cimò G., Marchese A., Germanà M.A. (2017). Microspore embryogenesis induced through in vitro anther culture of almond (*Prunus dulcis* Mill.). Plant Cell Tiss. Org. (PCTOC).

[59] Benjamini Y., Hochberg Y. (1995). Controlling the False Discovery Rate: A Practical and Powerful Approach to Multiple Testing. J. R. Stat. Soc. B.

[60] Anders S., Huber W. (2010). Differential expression analysis for sequence count data. Gen. Biol..

[61] Thimm O., Bläsing O., Gibon Y., Nagel A., Meyer S., Krüger P., Selbig J., Müller L.A., Rhee S.Y., Stitt M. (2004). MAPMAN: A user–driven tool to display genomics data sets onto diagrams of metabolic pathways and other biological processes. Plant J..

[62] Usadel B., Nagel A., Steinhauser D., Gibon Y., Bläsing O.E., Redestig H., Sreenivasulu N., Krall L., Hannah M.A., Poree F. (2006). PageMan: An interactive ontology tool to generate, display, and annotate overview graphs for profiling experiments. BMC Bioinform..

[63] Huang D.A.W., Sherman B.T., Lempicki R.A. (2009). Systematic and integrative analysis of large gene lists using DAVID bioinformatics resources. Nat. Protoc..

[64] Xia J., Benner M.J., Hancock R.E. (2014). NetworkAnalyst–integrative approaches for protein–protein interaction network analysis and visual exploration. Nucleic Acids Res..

