# Supplementary figures and images for: Gaining Insight into Exclusive and Common Transcriptomic Features Linked to Drought and Salinity Responses across Fruit Tree Crops
Source: Plants (Basel). 2020 Aug 19;9(9):1059. doi: 10.3390/plants9091059 (PMC7570245; doi:10.3390/plants9091059)

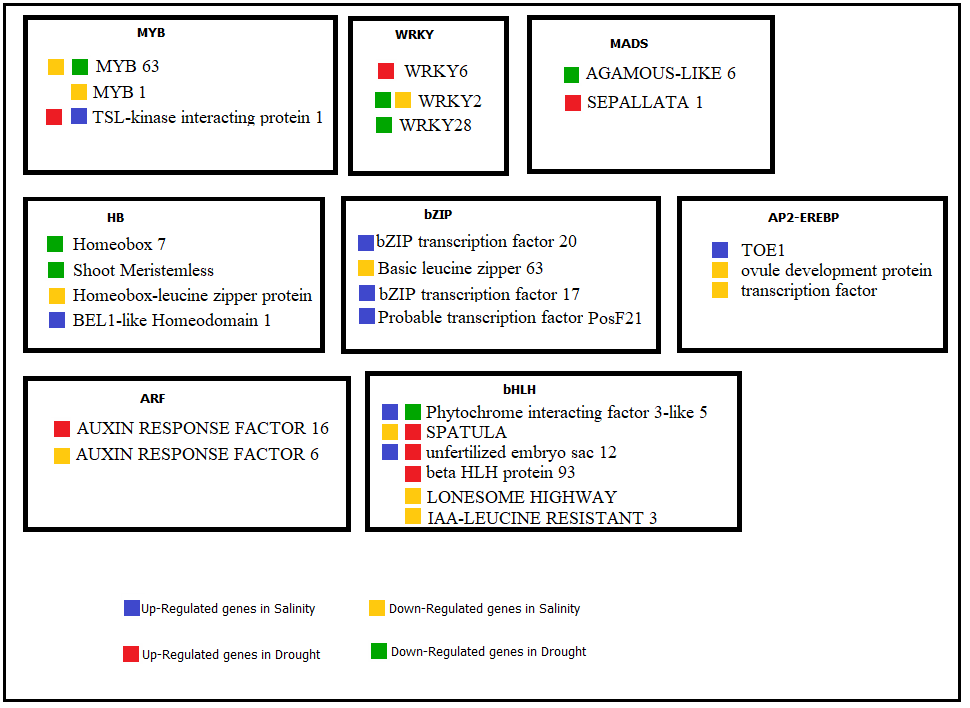

Supplement: Supplementary file 1 [file plants-09-01059-s001.zip › Supplementary_Files/Figure_S1.tif]

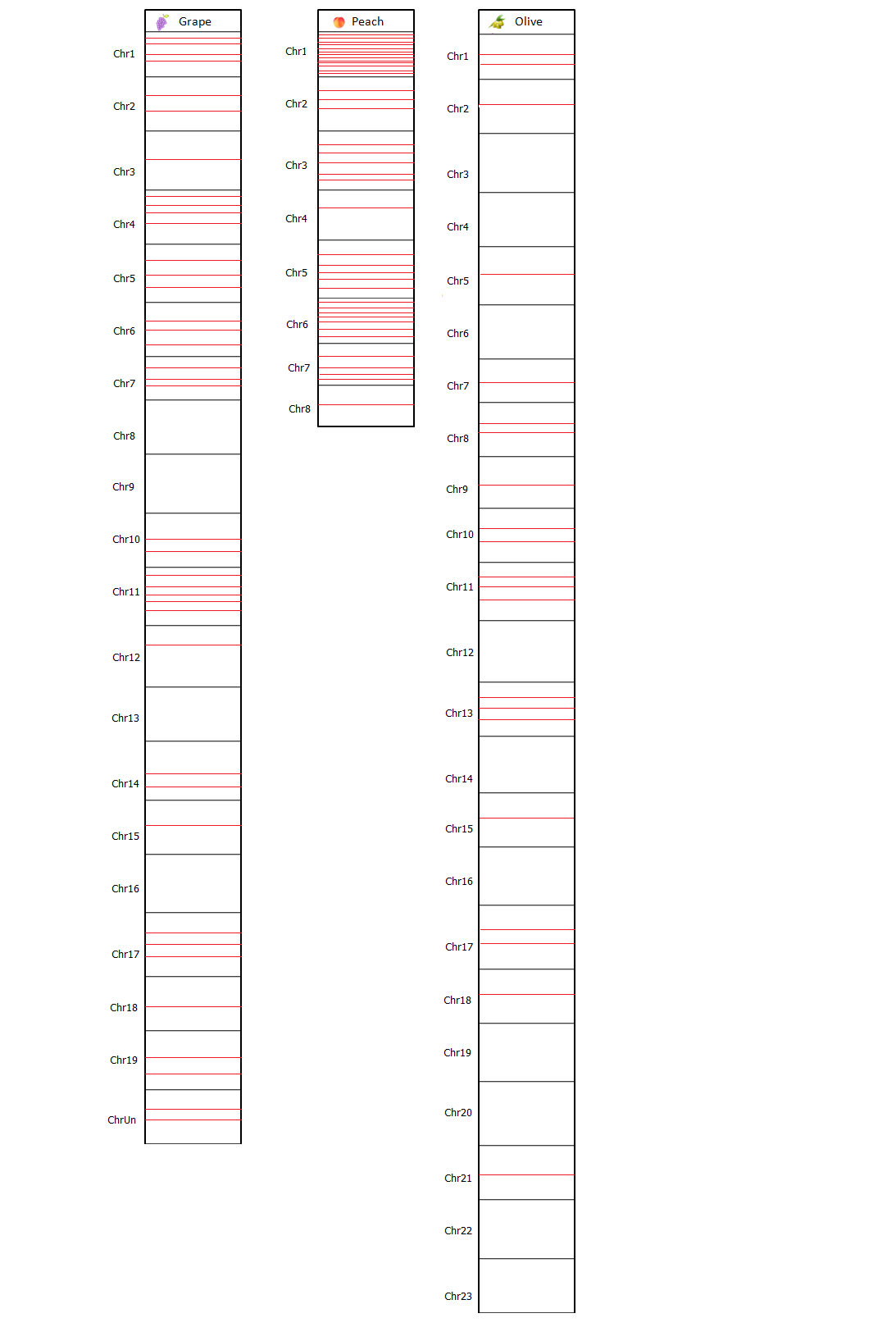

Supplement: Supplementary file 1 [file plants-09-01059-s001.zip › Supplementary_Files/Figure_S2.tif]

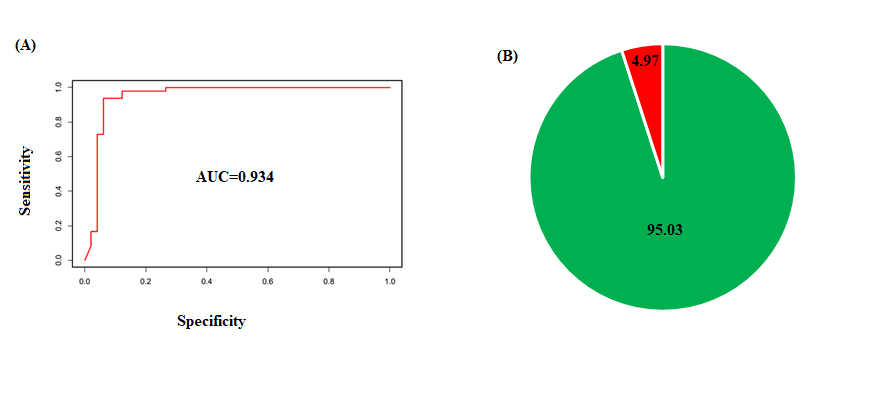

Supplement: Supplementary file 1 [file plants-09-01059-s001.zip › Supplementary_Files/Figure_S3.png]

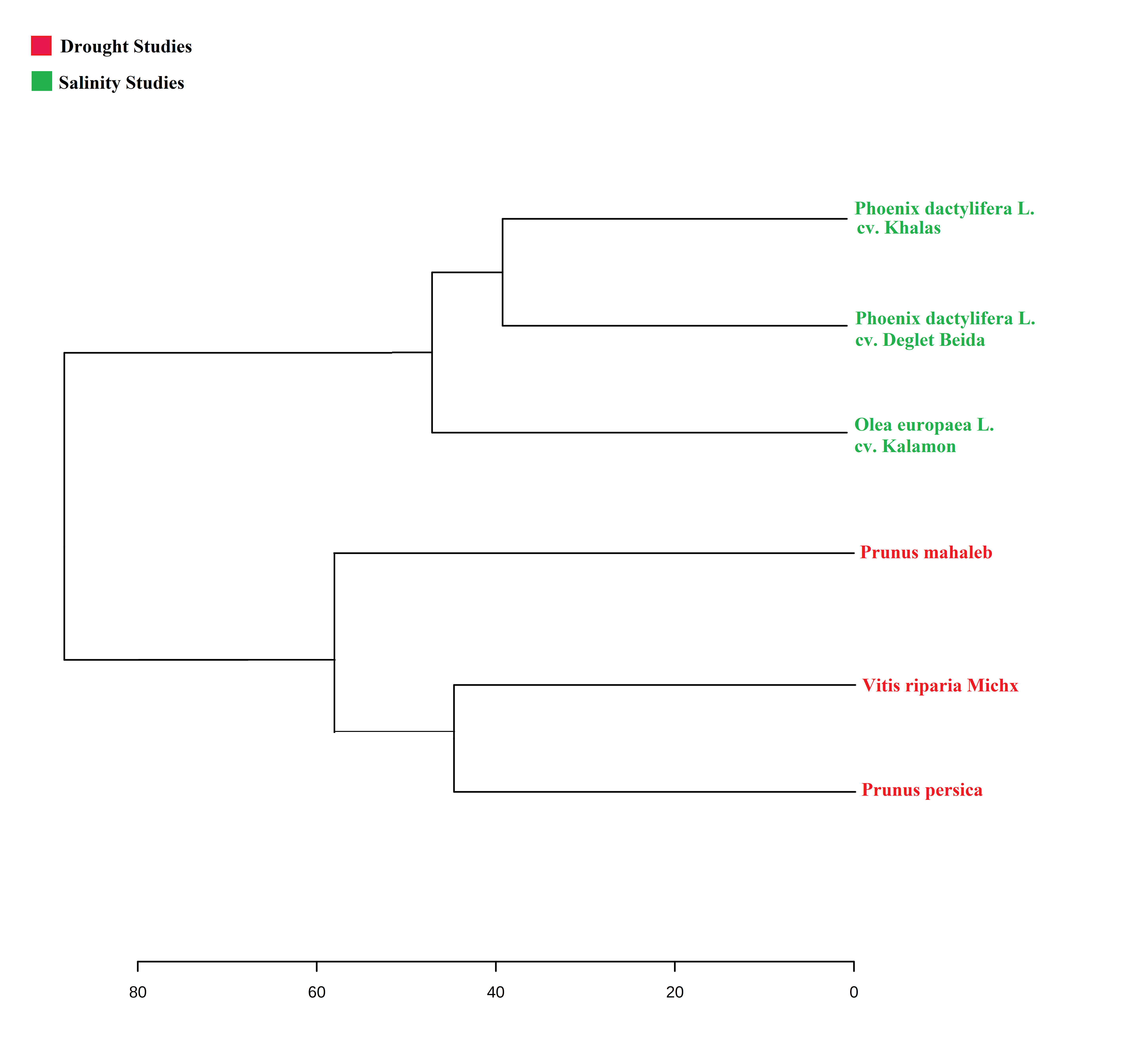

Supplement: Supplementary file 1 [file plants-09-01059-s001.zip › Supplementary_Files/Figure_S4.tif]
